# Does the Relationship Between Microelements (Copper, Zinc and Selenium) and Proinflammatory Proteins (IL-6, IL-8 and Tissue Factor) Have Diagnostic Value in Equine Medicine?

**DOI:** 10.3390/ijms262110429

**Published:** 2025-10-27

**Authors:** Wioleta Mojsym, Sylwester Kowalik, Agnieszka Chałabis-Mazurek, Iwona Janczarek, Witold Kędzierski

**Affiliations:** 1 Department of Biochemistry, Faculty of Veterinary Medicine, University of Life Sciences in Lublin, 20-033 Lublin, Poland; wioleta.mojsym@up.lublin.pl (W.M.); witold.kedzierski@up.lublin.pl (W.K.); 2 Department of Animal Physiology, Faculty of Veterinary Medicine, University of Life Sciences in Lublin, 20-033 Lublin, Poland; 3 Department of Pharmacology, Toxicology and Environment Protection, Faculty of Veterinary Medicine, University of Life Sciences in Lublin, 20-033 Lublin, Poland; 4 Department of Horse Breeding and Use, Faculty of Animal Sciences and Bioeconomy, University of Life Sciences in Lublin, 20-280 Lublin, Poland; iwona.janczarek@up.lublin.pl

**Keywords:** cytokine, exercise, horses, mares, pregnancy, trace elements

## Abstract

Some correlations between serum Cu, Zn and Se and cytokines have been reported in humans. Especially, the Cu:Zn ratio corresponded with inflammation. To date, relationships between microelements and proinflammatory proteins are poorly understood in horses. The aim of the study was to evaluate whether Cu, Zn and Se may influence turnover of IL-6, IL-8 and tissue factor (TF) in breeding and working horses. Blood samples obtained from 66 horses were analysed. There were 37 pregnant broodmares of different breeds, 13 barren broodmares and 16 race Thoroughbred horses. Serum Cu, Zn and Se concentration was determined using the gas flame atomic absorption spectrometry (GFAAS) method. Plasma IL-6, IL-8 and TF concentration was determined by the ELISA method. A coefficient correlation was carried out to compare the values of microelements studied with IL-6, IL-8 and TF using Pearson’s test. The values of IL-6 correlated significantly positively with Se and Cu:Zn ratio, IL-8 correlated positively with Cu and Cu:Zn ratio and negatively with Zn, and TF correlated positively with Cu, Cu:Zn ratio and Se. The Cu:Zn ratio varies significantly between horses, with high values occurring in horses with high levels of proinflammatory proteins, which may indicate the presence of a subclinical inflammatory process. The high variability of TF in the studied groups gives hope for the use of its determination in laboratory diagnostics of horses.

## 1. Introduction

Correlations between the levels of microelements, interleukins and Tissue Factor (TF) have, to date, been poorly studied in horses. Current research on this species is mostly focused on interleukins, without providing a deeper insight into the relationship between proinflammatory factors and minerals. This is a knowledge gap that the present study aims to address.

Some data indicate an association between the basal Cu:Zn ratio and a polymorphic variant of the equine IL-6 gene, where some variation of this gene correlates with a higher Cu:Zn ratio, although it does not influence IL-6 expression [1]. It has also been reported that IL-6 production in horses increases in the endometrium and placenta during pregnancy [2]. However, the potential relationship between microelement levels and the body’s ability to produce interleukins or TF during exercise or pregnancy remains poorly understood. This issue is particularly relevant in horses, which are used both for breeding and work purposes, and which frequently suffer from micronutrient deficiencies [3]. Among the known causes of elevated IL-6 levels in horses are the inflammatory response to acute exercise [4] and intestinal wall damage accompanying colic [5]. Prolonged transportation has also been identified as a stressor that significantly increases IL-6 and also IL-8 levels [6].

A number of scientific reports indicate the occurrence of relationships between the concentrations of microelements and cytokines in the blood plasma of humans and other mammals. For example, the serum copper (Cu) level correlated positively with interleukin 6 (IL-6) in women undergoing infertility treatment [7], and with interleukin 8 (IL-8) in a prospective birth cohort study [8]. In general, IL-6 stimulates inflammatory processes, helping to fight injuries and infections in body tissues; however, it can inhibit excessive inflammatory reactions and stimulate tissue regeneration. IL-8 is also a proinflammatory cytokine; however, unlike IL-6, it has a distinct target specificity for neutrophils [9]. Both of these cytokines have been demonstrated to play a regulatory role that is essential from the implantation phase, through their effects on the endometrium and embryo, to the regulation of hormonal activity [10].

Cu is known as a cofactor of a number of tissue enzymes, which indicates the element’s wide-ranging use in the body. Therefore, symptoms of copper deficiency can include muscle weakness, impaired coordination, fatigue and a reduced white blood cell count [11]. It is also known that the exposure of human monocytes to Cu ions leads to the substantial expression of tissue factor (TF) mRNA [12]. TF, also called Coagulation factor III, is a key factor in the initiation of blood clothing, in the transformation of fibrinogen to fibrin deposits and also in the synthesis of other cytokines, playing an important role during inflammation. TF synthesis is activated in humans in response to an endotoxemia [13]. In pregnancy, raised TF levels can lead to a hypercoagulable state, which is associated with a pregnancy disorder, so-called pre-eclampsia [14]. Blocking the synthesis or action of TF in mice prevents miscarriages [15]. Moreover, TF expression is stimulated by IL-6 [16] and IL-8 [17], the interleukins which correlate positively with Cu content in the body.

Zinc (Zn) is an essential element required for the proper functioning of about 3000 proteins in the body, responsible for, among others, immune and reproductive functions [18]. Supplementation with Zn has been shown to significantly increase the contents of IL-8 in cow’s milk [19], but in contrast, lower values of Zn with simultaneous higher values of IL-6 have been found in cytomegalovirus-infected pregnant women [20]. The plasma Cu:Zn ratio was associated with high plasma IL-6 levels in older adults, suggesting that the Cu:Zn ratio may be a marker of inflammation [21].

Selenium (Se) is a key factor in antioxidant status and the functioning of enzymes responsible for producing thyroid hormones that stimulate a basic metabolic rate. It is also an important element of the immune response, i.e., increasing IL-6 production [22]. The dependencies between the content of discussed microelements, inflammatory cytokines and TF mentioned above result from the participation of these microelements in gene expression and protein biosynthesis processes, in general. However, in some physiological and pathological conditions, there is a rapid increase in the concentration of interleukins or TF, while the content of microelements in the body remains stable at that time. Interleukins are secreted primarily in response to proinflammatory factors, like infections, injuries, fertilization, intense physical exercise, etc. and their concentration can increase substantially in a relatively short time measured in hours. In contrast, the content of microelements in the body depends on their supply in the diet and it does not change from day to day. For example, IL-6 production increases in muscles as a result of intense exercise [23].

As we have shown, there are research studies indicating the existence of a Cu:Zn ratio with proinflammatory cytokines both in humans and laboratory animals [21,24,25,26]. Thus, it was hypothesized that serum Cu:Zn ratio and Se levels may correlate with interleukins and TF in horses as well, indicating the occurrence of inflammation. The aim of the study was to evaluate whether selected microelements (Cu, Zn, Se) may influence the turnover of selected interleukins (IL-6, IL-8) and TF in breeding and working horses to confirm or reject the hypothesis about the existence of a relationship between the tested microelements and proinflammatory proteins. Additionally, the diagnostic significance of TF determination in horses was analysed, as there is no information on this subject so far. The study took into account the method of use and health condition of the horses.

## 2. Results

**Figure 1:**
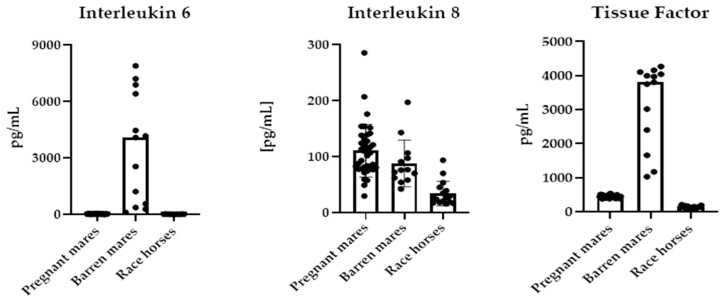
Plasma IL-6, IL-8 and TF concentration in barren mares (*n* = 13), pregnant mares (*n* = 37) and race horses (*n* = 16).

The results of IL-6, IL-8 and TF determination are presented in Figure 1.

The plasma IL-6 concentration was significantly higher in barren mares than in other groups of horses studied (Table 1). Plasma TF concentration was the highest in barren mares and the lowest in race horses. The values of IL-8 and Cu were lower whereas Zn level was higher in race horses than in both groups of mares. The serum Se concentration was higher in barren mares than in other groups of horses studied. In all studied horses, the values of IL-6 correlated significantly positively with Se and Cu:Zn ratio, IL-8 correlated positively with Cu and Cu:Zn ratio and negatively with Zn, and TF correlated positively with Cu, Cu:Zn ratio and Se (Table 2).

**Figure 2:**
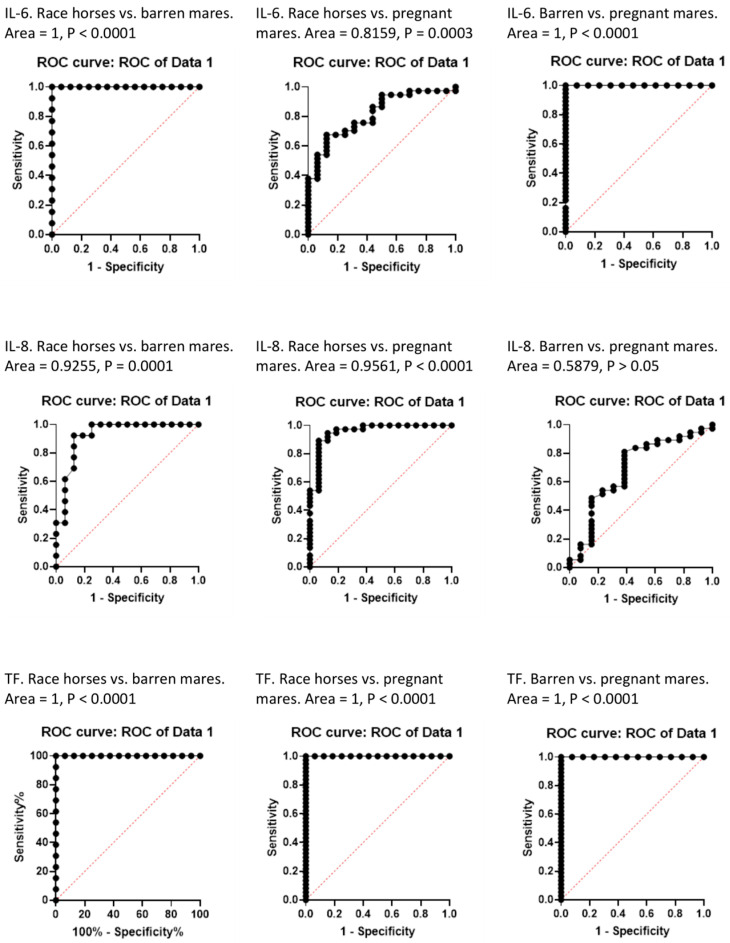
Results of IL-6, IL-8 and TF analysis in race horses (*n* = 16), pregnant mares (*n* = 37) and barren mares (*n* = 13) using Receiver Operating Characteristic (ROC) curves.

The ROC analysis showed statistically significant differences in IL-6, IL-8 and TF levels in the studied groups of horses, with the exception of IL-8 in barren and pregnant mares (Figure 2).

## 3. Discussion

The obtained results generally confirm the existence of correlations in horses between the content of Cu, Se and Cu:Zn ratio and the concentration of IL-6, IL-8 and TF in the blood, as shown in previous studies in humans and laboratory animals [7,8,12,22]. Moreover, the results of the ROC analysis confirmed statistically significant, high variability in IL-6 and TF concentration in race horses, pregnant mares and barren mares. In the study, a wide spectrum of horse breeds and type of use was included intentionally. It is known that equine IL-6 is coded by at least two genetic variants of genes, and the occurrence of a given gene variant is associated with the serum Cu:Zn ratio, regardless of the horse breed [1]. Therefore, it was important to include in the study a large number of genetically diverse horses to avoid the influence of a specific gene variant on the study results. It is also known that the main factors influencing plasma interleukin concentrations in healthy horses are physical exercise and pregnancy [2,4,23,27,28]. Therefore, three groups of horses were included in the study: trained race horses, pregnant mares, and unpregnant broodmares. It was not possible to find, as a control group, adult horses which were not used for sports, work and breeding purposes. Nevertheless, the selection of groups of horses for these studies allowed for the demonstration of relationships, among others, of Cu and the Cu:Zn ratio with the studied proteins. However, the content of trace elements in the diet of the studied horses was not the subject of research, as was the case in similar studies in humans and laboratory animals [21,24,25,26]. Furthermore, the age of the studied horses was not considered as a factor influencing the parameters studied. The horses in the study groups differed in both age and use. Young horses, aged 2–5 years, were in intensive racing training, while the broodmares were older and untrained. Therefore, the age of the studied horses was directly related to their use, and analysis of this aspect would have considered the two inextricably linked factors simultaneously. Furthermore, the occurrence of inflammation affecting the parameters we examined has been previously described in human studies, primarily in older subjects [21]. Among the horses we studied, there were no individuals that could be defined as old or geriatric; all horses fell into the “reproductive age” category.

Generally, the associations between Cu and interleukin levels in the body are diverse and ambiguous. For example, Cu is a component of superoxide dismutase, the enzyme that protects the body cells from oxidative damage. It has been reported that the activity of this enzyme decreases during inflammatory states [29]. Another key factor in the discussed relationships is the protein Copper Metabolism MURRI Domain 1 (COMMD1), which plays a crucial role in the regulation of Cu transmembrane transportation and control of inflammation. Genetically determined deficiency of this protein results in the excessive accumulation of copper ions in the liver, ceruloplasmin deficiency and low Cu concentration in the blood [30]. The expression of COMMD1 is decreased in a number of inflammatory disease states. On the other hand, this protein substantially increases the expression mRNA of IL-6 and IL-8 and the synthesis of these interleukins [31].

In turn, Se is incorporated into antioxidant enzymes such as glutathione peroxidase (GPX), which provides protection against reactive oxygen species. It has been reported that Se deficiency is connected with lower plasma IL-6 concentrations, whereas supplementation with Se leads to increased plasma IL-6 concentration in response to proinflammatory factors [32,33].

In contrast to the Cu- and Se-to-interleukins ratios discussed above, the results of the study did not confirm the positive relationship between Zn and interleukins that has been described in cows [19]. However, the horses involved in the study were very diverse in terms of Zn, IL-6 and TF contents. The studied group of race horses had unexpectedly high levels of Zn, two times higher than upper limit of the reference range, which amounted to 15–29 μmol/L [3]. Probably, the horses were over-supplemented with Zn, as indicated by both high Zn and low Cu levels, below the reference range (19–21 μmol/L). It is known that there exists an antagonism in the process of Zn and Cu absorption from the gut. High Zn supply could result in the inhibition of Cu absorption [34]. However, in this case it cannot be said to be zinc poisoning, since the horses did not show any clinical symptoms of poisoning, such as lameness or deterioration of the hoof condition; what is more, they took part in official races, which proves that they were in very good physical condition. Despite the excessive amount of Zn in these horses, plasma interleukins and TF concentrations remained very low. In the group of pregnant mares, plasma TF was significantly higher than in race horses. An elevated level of TF in bloodstream is typical for pregnancy [35]. On the other hand, barren mares showed extremely high levels of IL-6 and TF, with Cu and Zn concentrations at levels not different from those found in the other mares. A significant increase in plasma IL-6 concentration was reported in mares with chronic endometritis and subacute suppurative endometritis [36]. This finding suggests that the studied barren mares have developed subclinical endometritis, a common cause of difficulty with fertilization and implantation of the egg [37]. Subclinical endometritis is a particularly dangerous form of this condition because it can cause few or no symptoms but still lead to unfavourable long-term consequences. Moreover, due to the lack of clinical signs, this form of the disease is often overlooked by vets and can remain undiagnosed for a long time, increasing the risk of permanent changes in the uterus, ultimately leading to chronic infertility [37]. In turn it is surprising that in the mares in this group, the serum concentration of cortisol, a hormone regulating inflammatory reactions, was relatively low, not differing from the level observed in the other groups of the horses studied. As is known, cortisol is instrumental in regulating the inflammatory reaction and its blood concentration increases in response to serious illness [38]; however, subclinical metritis is not critical illness. Therefore, these interesting but ambiguous results do not definitively confirm that subclinical metritis may have been the cause of infertility in the studied mares.

Plasma TF has been less studied in horses, to date. Genetic studies conducted in mice have shown that TF functions depend largely on its site of secretion, and TF secreted in endothelial cells plays a greater role in the development of inflammatory processes than in the regulation of blood coagulation. In vitro studies have shown that proinflammatory factors directly affect endothelial cells, increasing TF production [39]. Proinflammatory cytokines and interleukins play a particular role in increasing TF levels [40]. In turn, in some disease models, TF in endothelial cells contributes to the expression of IL-6, suggesting its role in the development of inflammation independent of the activation of the coagulation cascade [39]. Experimental endotoxemia in humans induced profound, transient monocytopenia, along with activation of coagulation pathways. TF levels were elevated to varying degrees, depending on the monocyte subtype and the patient’s response to the inflammatory factor [13]. Literature data on the diagnostic significance of TF in horses are insufficient to interpret the obtained results. Nevertheless, the results of our study confirm the existence of a correlation between the Cu:Zn ratio and the concentration of IL-6, IL-8 and TF in blood plasma, thus confirming the hypothesis about the possibility of using the Cu:Zn ratio as a marker of inflammation not only in humans [21,41] but also in horses.

## 4. Materials and Methods

### 4.1. Horses

Blood samples obtained from 66 horses were used in the study. They were samples collected from 50 broodmares and 16 Thoroughbred horses trained on a racetrack. Samples were taken by veterinarians directly supervising the health status of these horses as a part of health examination. Therefore, according to the Polish regulations regarding experiments on animals and the European directive EU/2010/63, obtaining the special approval of the ethical committee for blood sampling, qualified as non-experimental clinical veterinary practices, was not necessary. The owners of the study animals were informed about the purpose of blood collection and gave their verbal consent to the use of the collected samples for research studies and to the publication of the study results.

All studied broodmares were maintained in breeding farms. They were kept in stables with access to paddocks and fed with hay, oats and commercial feed for breeding mares. They belonged to different breeds and types of use: Purebred Arabian mares representing sport breed (22 individuals), Hucul horses (16 mares) belonging to primitive horses and those representative of working horses (12 crossbreed mares). Of the 50 mares tested, 37 were in the last three months of pregnancy with known mating day and 13 individuals representative of the sport breed had been barren since the previous breeding season, despite repeated inseminations and veterinary treatment. All studied mares, pregnant and barren, were in good and very good body condition, without any clinical signs of health disturbances at the time of sampling.

The 16 Thoroughbred horses tested were maintained and trained on a race track. This group included 13 stallions and 3 mares aged 2–5 years. The horses were fed and taken care of in the manner used for race horses. They received individually calculated rations of oats, hay and mineral–vitamin high-energy feed concentrate. All the horses participated in official races during the race season. At the day of blood sampling, none of the horses showed any signs of health disturbances.

### 4.2. Blood Sampling and Analyses

Blood sampling took place from February to April of one calendar year, always in the morning, from horses at rest. After jugular vein venipuncture, 9 mL of blood was collected into a centrifuge serum tube and another 5 mL in a tube containing 3-potassium EDTA and aprotinin (BD Vacutainer, Plymouth, UK). The filled serum tubs were left for approximately two hours until the blood clotted completely, whereas filled EDTA tubs were immediately cooled down by putting them into a water bath at a temperature of 4 °C for about 30 min. Then, all tubes were centrifuged at 1000× *g* for 20 min for serum separation or for 15 min for plasma separation. The obtained serum and plasma samples were frozen and stored at −80 °C until further analysis.

Serum samples were used to determine the concentrations of Cu, Zn and Se by the gas flame atomic absorption spectrometry (GFAAS) method. Detailed information concerning the method of Cu and Zn determination are described by [42]. The determination process was controlled by analysing a series of samples from the certified reference material Seronorm Trace Elements Serum L-2 (Sero Inc., Billngstad, Norway). The Se concentration was determined according the modified Neve and Molle method [43]. The blood serum samples were diluted to a 1:3 ratio with a matrix modifier: 0.5 g/L Cu (II) acetate, 1.0 g/L Mg (II) nitrate, 0.15% (*m*/*v*) TritonX-100 in 2% (*m*/*v*) HNO_3_. Validation parameters for the determination of Se in serum blood comprised the following: detection limit, quantification limit, recovery and repeatability/precision of the method were 3.5 µg/L, 7.0 µg/L, 93.6% and 3.2%.

Serum cortisol concentration was determined using the immune-enzymatic Cortisol ELISA kit (DRG Instruments GmbH, Marburg, Germany). The sensitivity of this kit amounts to 1.3 ng/mL and the range is between 1.3 and 800 ng/mL.

Plasma concentrations of IL-6, IL-8 and TF were determined using immune-enzymatic methods (Horse IL-6, IL-8, TF ELISA Kits, FineTest, Wuhan, China). For the IL-6 test, the range, sensitivity and linearity amounted to 3.125–200 pg/mL, 1.875 pg/mL and 82–100%; for IL-8, 15.625–1000 pg/mL, 9.375 pg/mL and 85–99% and for TF: 7.813–500 pg/mL, 4.688 pg/mL and 82–94%, respectively. For each test, absorbance was measured at 450 nm using a Multiskan reader (Labsystem, Helsinki, Finland) supported by GENESIS V 3.0 software. Each sample was measured in duplicate. The average value of the two determinations was taken for further statistical analysis.

### 4.3. Statistical Analysis

Statistical analysis was performed using the software package GraphPad Prism^TM^, version 9.5.0 (Graph Pad Software, La Jolla, CA, USA). All data were tested for normality of distribution by the Shapiro–Wilk test. A normal distribution was confirmed for Cu, Zn, Se, cortisol and IL-8 data (W > 0.9514, *p* > 0.05). Thus, they were analysed using a one way ANOVA and Tukey’s test as the post hoc comparison of analysed results, quantified as means with standard deviations (±SD). The values of IL-6 and TF did not pass the test for normality of distribution (W = 0.85, *p* = 0.004 and W = 0.82, *p* = 0.0006, respectively), thus they were analysed using a non-parametric Kruskal–Wallis test, and the results were quantified as medians and Quartile deviations (QD). The differences between the means/medians in the studied groups of horses were considered significant at *p* ≤ 0.05. The coefficient correlation was carried out to compare the values of microelements studied with IL-6, IL-8 and TF using Pearson’s test or a nonparametric Spearman correlation test. Received Operating Characteristic (ROC) analysis was also used to evaluate the accuracy of IL-6, IL-8 and TF as classifiers of some physiological or pathological states of the studied horses.

## 5. Conclusions

The Cu:Zn ratio varies significantly between horses, with high values occurring in horses with high levels of proinflammatory proteins, which may indicate the presence of subclinical inflammation. Regardless of the relationships between the studied microelements and proinflammatory proteins, IL-6, IL-8 and TF concentrations show significant variation among fit horses, pregnant mares and barren mares, which raises the possibility of using these parameters as indicators of specific physiological and/or pathological states. TF was found to be a factor with high variability in horses depending on their health status and type of use, which was described for the first time. However, further research is required to confirm the relationship between TF levels and health status, pregnancy, or infertility in mares.

## Figures and Tables

**Table 1:** Plasma IL-6, IL-8, TF, cortisol and serum Cu, Zn and Se concentrations in the studied horses.

Parameters	Pregnant Mares *n* = 37	Barren Mares *n* = 13	Race Thoroughbreds *n* = 16	*p* Value
Medians ± QD				
IL-6 [pg/mL]	15.6 ± 8.60 ^a^	4085 ± 2793 ^b^	8.75 ± 3.45 ^a^	≤0.0001
TF [pg/mL]	486 ± 135 ^a^	3804 ± 1155 ^b^	142 ± 36.9 ^c^	≤0.0001
Means ± SD				
Il-8 [pg/mL]	103 ± 56.5 ^a^	88.0 ± 39.9 ^a^	35.1 ± 19.8 ^b^	≤0.0001
Cortisol [ng/mL]	93.1 ± 50.9	101 ± 60.2	73.4 ± 12.7	≤0.0001
Cu [µmol/L]	17.9 ± 4.21 ^a^	20.9 ± 3.04 ^a^	7.84 ± 1.80 ^b^	≤0.0001
Zn [µmol/L]	10.2 ± 1.22 ^a^	9.01 ± 1.34 ^a^	70.6 ± 51.5 ^b^	≤0.0001
Se [µmol/L]	0.77 ± 0.42 ^a^	1.49 ± 0.60 ^b^	0.84 ± 0.11 ^a^	≤0.0001
Cu:Zn ratio	1.78 ± 0.44 ^a^	2.37 ± 0.45 ^b^	0.11 ± 0.09 ^c^	≤0.0001

^a–c^ values marked with different letters differ significantly.

**Table 2:** Correlations between Il-6, IL-8, TF and the microelements in the studied horses (*n* = 66).

Correlation Coefficient	Cu	Zn	Cu:Zn Ratio	Se
IL-6	0.342	−0.213	0.418 **	0.433 ***
IL-8	0.422 ***	−0.415 ***	0.490 ****	0.079
TF	0.487 ***	−0.327	0.571 ****	0.541 ****

Values statistically significant at: ** *p* ≤ 0.01, *** *p* ≤ 0.001, **** *p* ≤ 0.0001.

## Data Availability

The data presented in this study are available on request from the corresponding author due to the author’s ongoing work and further analysis.
